# Resistive switching and battery-like characteristics in highly transparent Ta_2_O_5_/ITO thin-films

**DOI:** 10.1038/s41598-023-40891-2

**Published:** 2023-08-31

**Authors:** Darshika Khone, Sandeep Kumar, Mohammad Balal, Sudipta Roy Barman, Sunil Kumar, Abhimanyu Singh Rana

**Affiliations:** 1 Centre for Advanced Materials and Devices, School of Engineering and Technology, BML Munjal University, Gurgaon, 122413 India; 2 Department of Physics, Indian Institute of Technology Delhi, New Delhi, 110016 India; 3 UGC-DAE Consortium for Scientific Research, Indore, 452001 India

**Keywords:** Materials science, Nanoscience and technology, Optics and photonics, Physics

## Abstract

Highly transparent resistive-switching (RS) devices were fabricated by growing amorphous tantalum pentoxide (a-Ta_2_O_5_) and indium tin oxide (a-ITO) thin films on barium-borosilicate glass (7059) substrates, using electron beam evaporation. These layers exhibited the transmittance greater than ~ 85% in the full visible region and showed RS behavior and battery-like IV characteristics. The overall characteristics of RS can be tuned using the top electrode and the thickness of a-Ta_2_O_5_. Thinner films showed a conventional RS behavior, while thicker films with metal electrodes showed a battery-like characteristic, which could be explained by additional redox reactions and non-Faradaic capacitive effects. Devices having battery-like IV characteristics showed higher enhanced, retention and low-operation current.

## Introduction

Transparent resistive-switching (RS) devices have attracted significant scientific attention for developing invisible circuits, ultra-low power electronic devices, sensors, and transparent electronics^1–10^. Current research on transparent-conducting-oxides (TCO) requires a delicate balance between high electrical conductivity and optical transparency in the visible spectrum. Moreover, there is a need of embedding other circuitry elements on TCO that can perform other information processing and storage functionalities. Especially, the two-terminal RS device is promising to overcome the fundamental limitations of circuit complexity, scaling and power consumption^8,11–15^. In a typical RS device, the resistance is changed between low and high resistive states in a reversible and non-volatile manner under the application of electric fields and currents. Furthermore, the multilevel RS can also have intermediate-resistive-states (IRS) that can be stabilized in a single device using voltage, compliance-current and temperature, which could play a pivotal role for high-density storage^16–22^. Another related behavior is memristive type, where the resistance value is continuously modified with the history of applied voltage and current that can mimic the condition in the brain, in which the electrical connections between two neurons become stronger every time the connection is addressed^21,23–27^. Both memristive and multilevel switching characteristics are essential for the development of neuromorphic computing. Indeed, the same two-terminal RS devices (in capacitator geometry) can also be leveraged to provide the power locally, also called nanobatteries^28,29^ for increasing the portability and efficiency. High dielectric metal-oxides such as hafnium-oxide (a-HfO_x_), tantalum-oxide (a-Ta_x_O_y_) and yttrium oxide (a-Y_2_O_3_) could be very promising materials for developing transparent-resistive-random-access-memory (T-RRAM) and memristive devices, that can be grown at low temperature^22,30–36^. Tantalum-oxides (a-Ta_2_O_5_) grown by various physical vapor deposition methods are highly promising as highly insulating transparent films can be grown at lower temperatures. Therefore, it would be interesting to elucidate the role of film thickness, electrode material, Joule’s heating, and whether or not transparent conducting oxide like indium tin oxide (ITO) can be effective integrated with tantalum-oxide for T-RRAM or transparent memristors. Here, we have found that some intermediate thickness regime showed a new kind of battery-like IV characteristics with enhanced endurance, retention and extremely low leakage current that is important for the low-power devices. We propose a non-Faradaic capacitive (NFC) effect could be responsible for this behavior. The opposing internal field helps in restricting the leakage current and improve the operation power in these devices. This study opens the opportunities of incorporating the concepts of nanobatteries in Ta_2_O_5_ based memristors and RS devices, and pose further questions related to the underlaying mechanisms of NFC.

## Result and discussions

**Figure 1:**
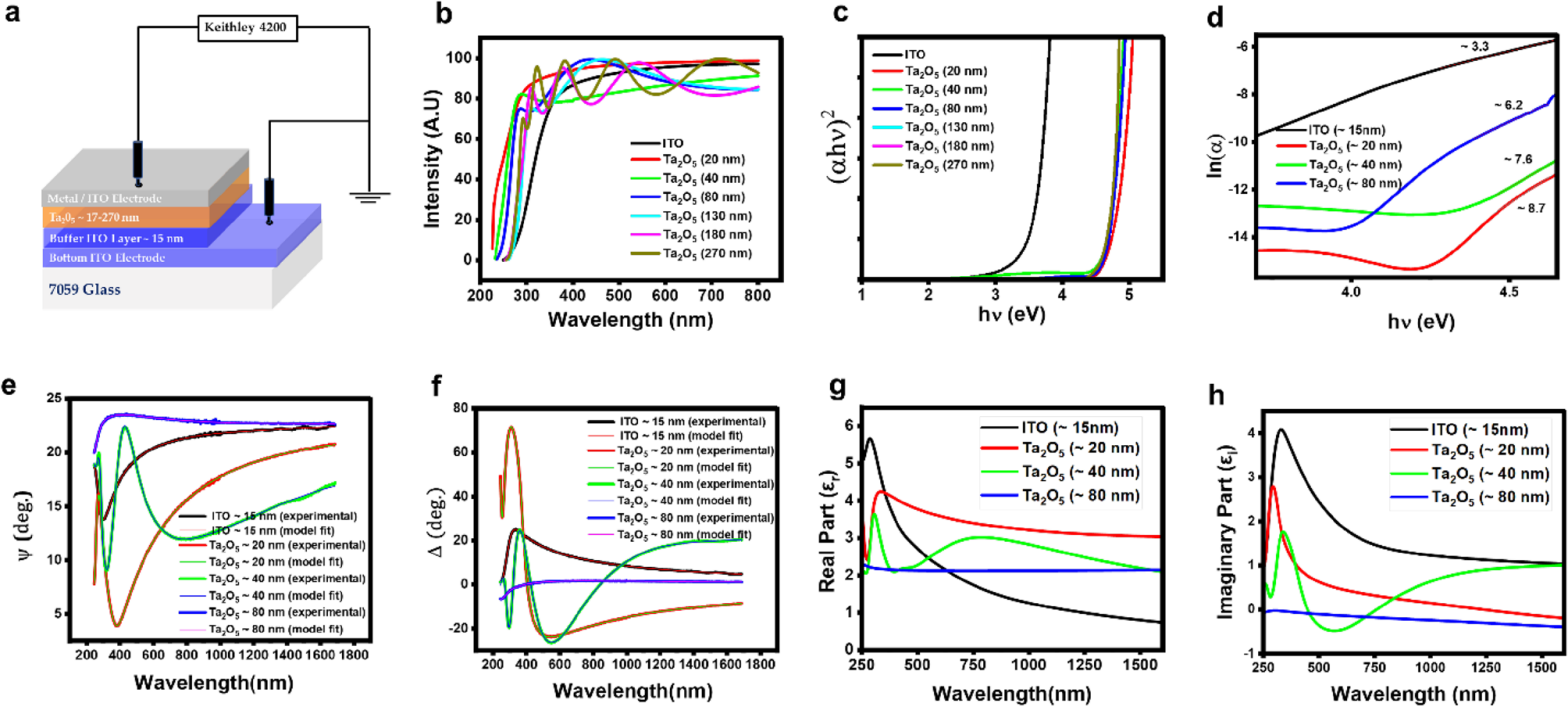
(**a**) schematic diagram of the resistive switching devices, (**b**) UV–Vis. Transmission vs Wavelength of individual layers, (**c**) Tauc plots to extract the bandgaps of individual layers, (**d**) The logarithmic variation of the absorption coefficient versus photon energy for ITO (~ 15 nm), Ta_x_O_y_ (~ 20 nm, ~ 80 nm), (**e**–**f**) The spectra of ellipsometric parameters, Δ (a) and ψ (b) verses wavelength (λ), (**g**,**h**) Real part (ε_r_) and Imaginary part (ε_i_) of the dielectric constant versus wavelength (λ).

Figure 1a illustrates the schematic diagram of resistive switching (RS) devices. To fabricate these devices, a highly transparent bottom electrode layer of amorphous Indium Tin Oxide (a-ITO) was first deposited on barium-borosilicate glass (Corning 7059 glass) using electron beam evaporation (EBE). After masking a region for the bottom contact, fresh layers of a-ITO of ~ 15 nm thickness and a-Ta_2_O_5_ of distinct thicknesses (~ 20 nm, ~ 40 nm, ~ 80 nm, ~ 130 nm, ~ 180 nm, ~ 270 nm) were deposited without breaking the vacuum to ensure a fresh interface. The EBE of a-ITO and a-Ta_2_O_5_ were performed using 99.999% pure granules of these materials under the oxygen partial pressure of ~ 2 × 10^–5^ Torr at substrate temperature ~ 200 °C and electron beam current of ~ 40 mA. The shadow mask was used to fabricate top metal electrodes where area of circular metal electrodes was found to be ~ 100 μm^2^. The thickness and roughness of these thin films were confirmed using the stylus profilometer and ellipsometry.

UV–visible spectroscopy and ellipsometry are used to investigate the optical properties of these films. Figure 1b shows the transmittance spectra measured over the wavelength range of 250–800 nm of a-ITO and a-Ta_2_O_5_ of different thicknesses. The transmission of individual layers of a-Ta_2_O_5_ oxide and a-ITO is more than ~ 85% in the full visible region, and ~ 94% @ 550 nm wavelength. The Fabry–Perot oscillations in the UV–visible spectra are seen with increasing the film thicknesses, indicating high quality of these films and confirm the layer-by-layer smooth growth. The absorption coefficient  of the film can be derived from the equation , where T, R and *d* are transmittance, reflectance, and thickness respective. It can be further simplified as  for negligible reflectance. The bandgaps of these films were extracted from UV–Visible spectra using the Tauc plots, as shown in Fig. 1c, using the equation  where $h, v$ and ${E}_{g}$ are Plank’s constant, light frequency and bandgap respectively. The optical bandgap value of a-ITO is ~ 3.6 eV and a-Ta_2_O_5_ is ~ 4.5–4.8 eV, which is consistent with the literature values^37,38^ within the limitations of extracting bandgap using Tauc plots. The a-Ta_2_O_5_ films are highly insulating and transparent that indicate the very low oxygen vacancies and defects. To further understand the defects, the Urbach energy (E_u_) can be extracted from the UV–Vis spectroscopy that indicate the band-tailing^39^ in amorphous this film due to the presence of disorders^39^. It can be derived from the exponential broadening of the absorption edge using the relation $\alpha ={\alpha }_{0}\mathrm{exp}\frac{hv}{{E}_{u}}$. Figure 1d illustrates ln (*α*) *vs* h*ν* plots and the corresponding slopes are mentioned on the corresponding curves. The inverse of the slope gives the approximate values of E_u_ for ITO ~ 200 meV and Ta_2_O_5_ ~ 115 meV (for ~ 20 nm), 131 meV (for ~ 40 nm) and ~ 161 meV (for ~ 80 nm). The increase in the Urbach energy with thickness indicate the formation of higher oxygen vacancies as the films are grown thicker. This is also consistent with the slight decrease in the overall transmission of these films as the thickness increases.

The optical and dielectric properties of these films were further investigated using the variable angle spectroscopic ellipsometer (VASE) model developed by J. A. Woollam, Inc. The ellipsometry measurements were carried out at an incident angle of 70° (close to Brewster's angle for these samples) at the wavelengths (λ) ranging from 250 to 1650 nm with energy resolution of ~ 0.01 eV. These results are shown in Fig. 1e–h. Here, the change in the polarization (ρ) of incident light is measured after the reflection from the sample surface which can be expressed as $\rho = {r}_{p}/{r}_{s}$ , where ${r}_{s}$ and ${r}_{p}$ are the amplitude of *s*- and *p-* components reflected waves having oscillations parallel and perpendicular to the sample surface, respectively^40^. In a typical measurement, the complex reflectance ratio $\rho =\frac{{r}_{p}}{{r}_{s}}=\mathrm{tan}(\psi ).\mathrm{exp }(i\Delta )$, where $\mathrm{tan}(\psi )$ is amplitudes ratio and $\Delta$ is the phase difference i.e $\rho = \frac{\left|{r}_{p}\right|\mathrm{exp}(i{\delta }_{p})}{\left|{r}_{s}\right|\mathrm{exp}(i{\delta }_{s})}$. The parameters ($\psi , \Delta )$ can be calculated using Fresnel equations using the iterative model. Here, we have used a two-layer optical model suitable for tantalum oxide thin films^40^, and the fitted graphs are shown in Fig. 1e,f. It is possible to see that the modelled fit spectra and experimental spectra coincide almost perfectly. These results indicate that $\Delta$ is more sensitive to variations in film thickness than $\psi$ throughout the range of thicknesses. Specifically, the thicker film and the thinner film showed little variation in the $\psi$ vs. wavelength curves, although the equivalent curves for $\Delta$ showed a huge variation. The film deposition parameters would have an impact on the "$\psi , \Delta$" values. Various optical parameters such as refractive index (*n*), extinction coefficient (*k*), film thickness, and roughness can be extracted as fitting parameters from Fig. 1e,f, as shown in the Supplementary Fig. S1c,d. The values of *n* and *k* are used to estimate the real and imaginary components of dielectric constants using the relation ${\varepsilon }_{r}={n}^{2}-{k}^{2}$ and ${\varepsilon }_{i}=2nk$ respectively, as shown in Fig. 1g,h as a function of wavelength. The imaginary part of the dielectric material corresponds to the dissipation or loss of incident photon energy inside the material. The refractive index (n) represents the part of the light that gets transmitted through the material without getting absorbed and depends on the chemical composition of the material. The extinction coefficient (k) depends on the defects present in the material. For a good dielectric material, imaginary part (or dissipation) should be as small as possible and real part should be large. Apparently, the large change in parameters ($\psi , \Delta )$ and the dielectric constants (${\varepsilon }_{r}, {\varepsilon }_{i})$ could be seen near the wavelengths (250–500 nm) near bandgap due to the band-to-band transitions^40^.

The accuracy of the fitting^40,41^ can be quantified as the root mean squared error $MSE= \sqrt{\frac{1}{3m-1}}\sum_{i=1}^{m}\left[{\left({P}_{Ei}-{P}_{Gi}\right)}^{2}+{\left({D}_{Ei}-{D}_{Gi}\right)}^{2}+{{K}_{Ei}-{K}_{Gi})}^{2}\right]\times 1000$, where $P=\mathrm{cos}\left(2\psi \right)$; $D=\mathrm{sin}\left(2\psi \right)\mathrm{cos}\left(\delta \right)$; $K=\mathrm{sin}\left(2\psi \right)\mathrm{sin}\left(\delta \right)$; *m* is the number of measured wavelengths and *i* is the iteration number. MSE values are found to be ~ 1 for all the thin films.

**Figure 2:**
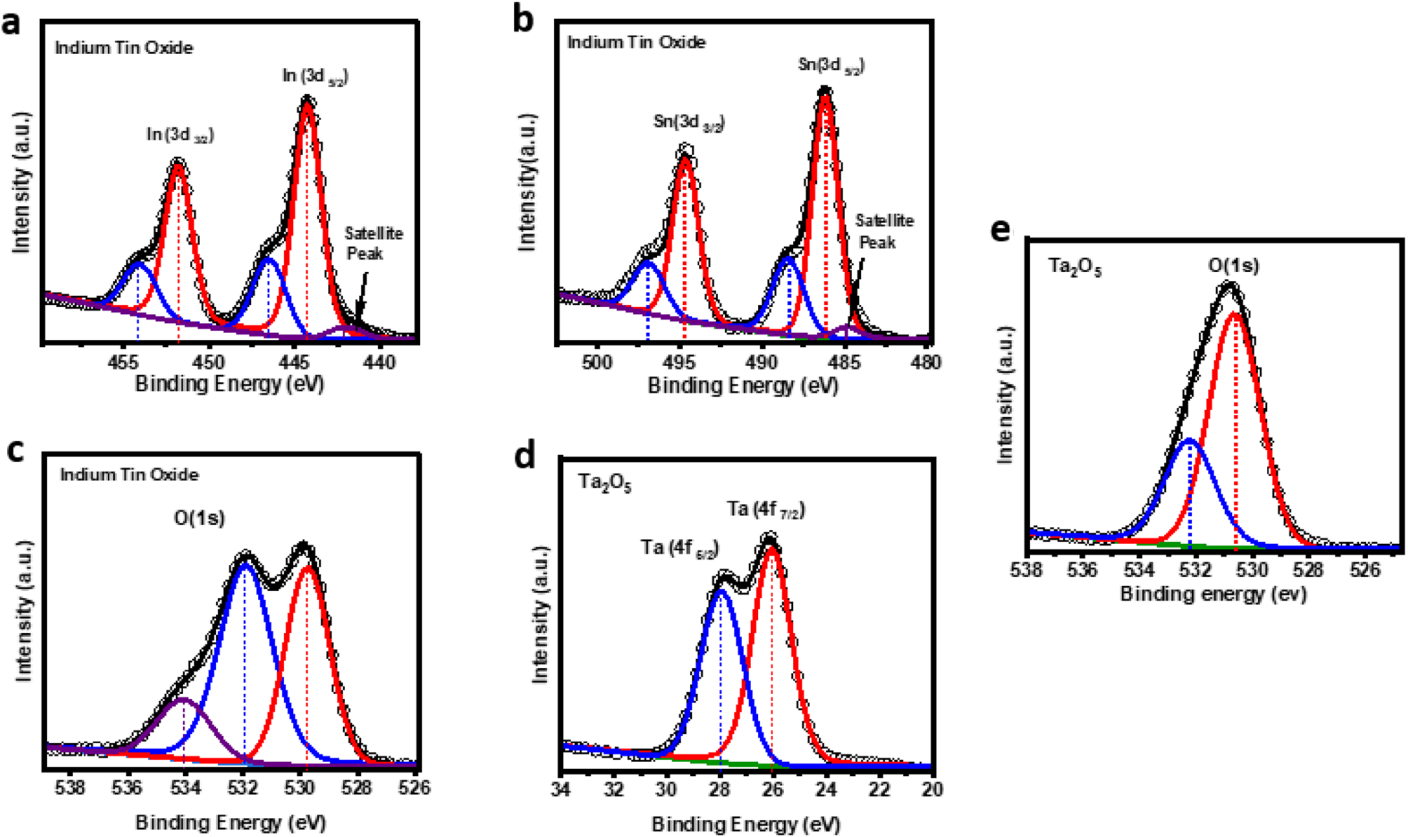
(**a**–**c**) XPS results of ITO thin films, (**a**) indium (In) peaks related to 3d_3/2_ and 3d_5/2_ orbitals, (**b**) Tin (Sn) peaks related to 3d_3/2_ and 3d_5/2_ orbitals, (**c**) Oxygen (O) peak related to 1 s orbitals. (**d**,**e**) XPS results of Ta_2_O_5_ thin films, (**d**) tantalum (Ta) peaks related to 4f_7/2_ and 4f_5/2_, (**e**) Oxygen (O) peak related to 1 s orbitals.

X-ray photoelectron spectroscopy (XPS) measurements were performed using a hemispherical electron energy analyzer with 20–50 eV pass energy with an energy resolution of ~ 1 eV at a base pressure of ~ 2 × 10^−10^ mbar. A non-monochromatic Al_Kα laboratory X-ray source was operated at ~ 196 W (14 mA and 14 kV). The specimens were mounted using a conducting carbon tape on a specially designed sample station^42^. The XPS data was analyzed with xpspeak41 software. The multiple peaks in XPS spectra were fitted using Gaussian function after Tougaard type background subtraction. The position of sp^3^ carbon 1 s peak is taken as a standard (binding energy 284.6 eV) to compensate for any charge-induced shifts. The spin- orbit splitting for Indium (In) and Tin (Sn) are 7.54 eV and 8.47 eV, respectively. The XPS spectra of a-ITO layer shows peaks around ~ 451.79 eV and ~ 454.12 eV related In 3d_3/2_ orbitals and ~ 446.49 eV and ~ 444.24 eV related to In 3d_5/2_ orbitals, as shown in Fig. 2a. On the other hand, Sn peaks 3d_3/2_ appears around ~ 496.96 eV and ~ 494.70 eV and Sn 3d_5/2_ peaks around ~ 488.40, ~ 486.18, as shown in Fig. 2b. The peaks ~ 442.09 eV and ~ 484.89 eV are satellite peak due to non-monochromatic x-ray source. The binding energy of In and Sn peaks indicate the valency of these elements are 3^+^ and 4^+^ respectively, that confirms the mixture of In_2_O_3_ and SnO_2_. Oxygen (O) 1 s orbital peak coincides well with ~ 529.7, ~ 531.8, ~ 534.0 with expected binding energy of In_2_O_3_ and SnO_2_ as shown in Fig. 2c. Similar results were observed in the literature paper^43,44^. The peak of In 3d_5/2_ at ~ 446.49 and O 1 s at ~ 534.0 peak may be attributed due to free hydroxyl groups which is possibly due to environmental moisture trapped in the film surface. XPS spectra of freshly grown a-Ta_2_O_5_ layer ~ 17 nm shows two sharp peaks at ~ 26.0 eV and ~ 27.9 eV in Fig. 2d related to tantalum (Ta) 4f_7/2_ and 4f_5/2_ orbitals, respectively. The oxygen (O) 1 s orbital peak is seen around ~ 530.63 eV and 532.22 eV in Fig. 2e. The valency is confirmed by the binding energy position of Ta 4f_7/2_^45–48^. Therefore, the XPS results clearly indicate the stochiometric nature of Ta_2_O_5_^46,49,50^. The similar results of XPS were obtained on thicker films which also confirms the similar stoichiometry surfaces of both the films, but this technique has a limitation and only films up to ~ 2 nm can be probed below the surface.

**Figure 3:**
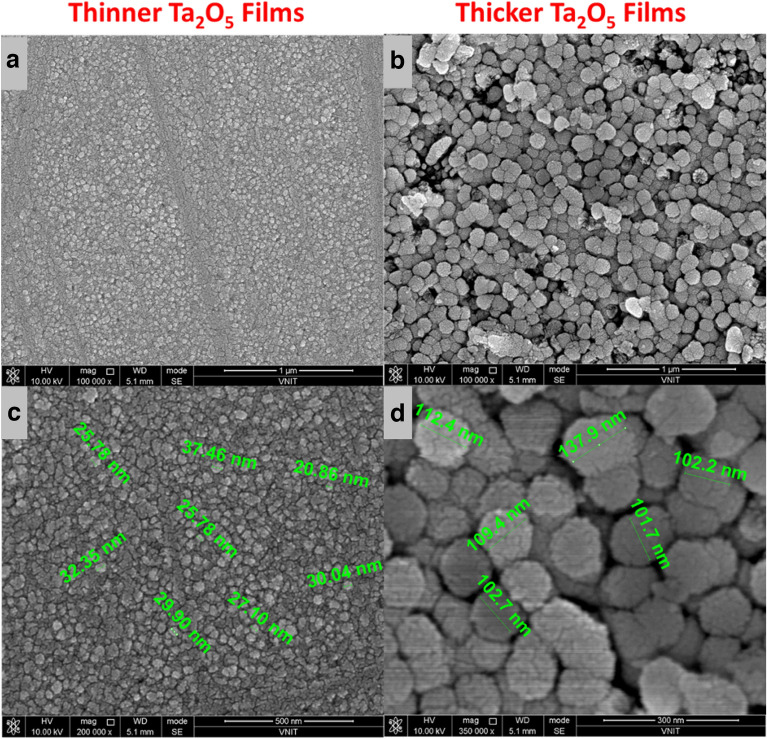
(**a**,**b**) FESEM topographic images of thinner and thicker films grown on ITO coated substrates (**c**,**d**) their approximate distribution of particle size.

**Figure 4:**
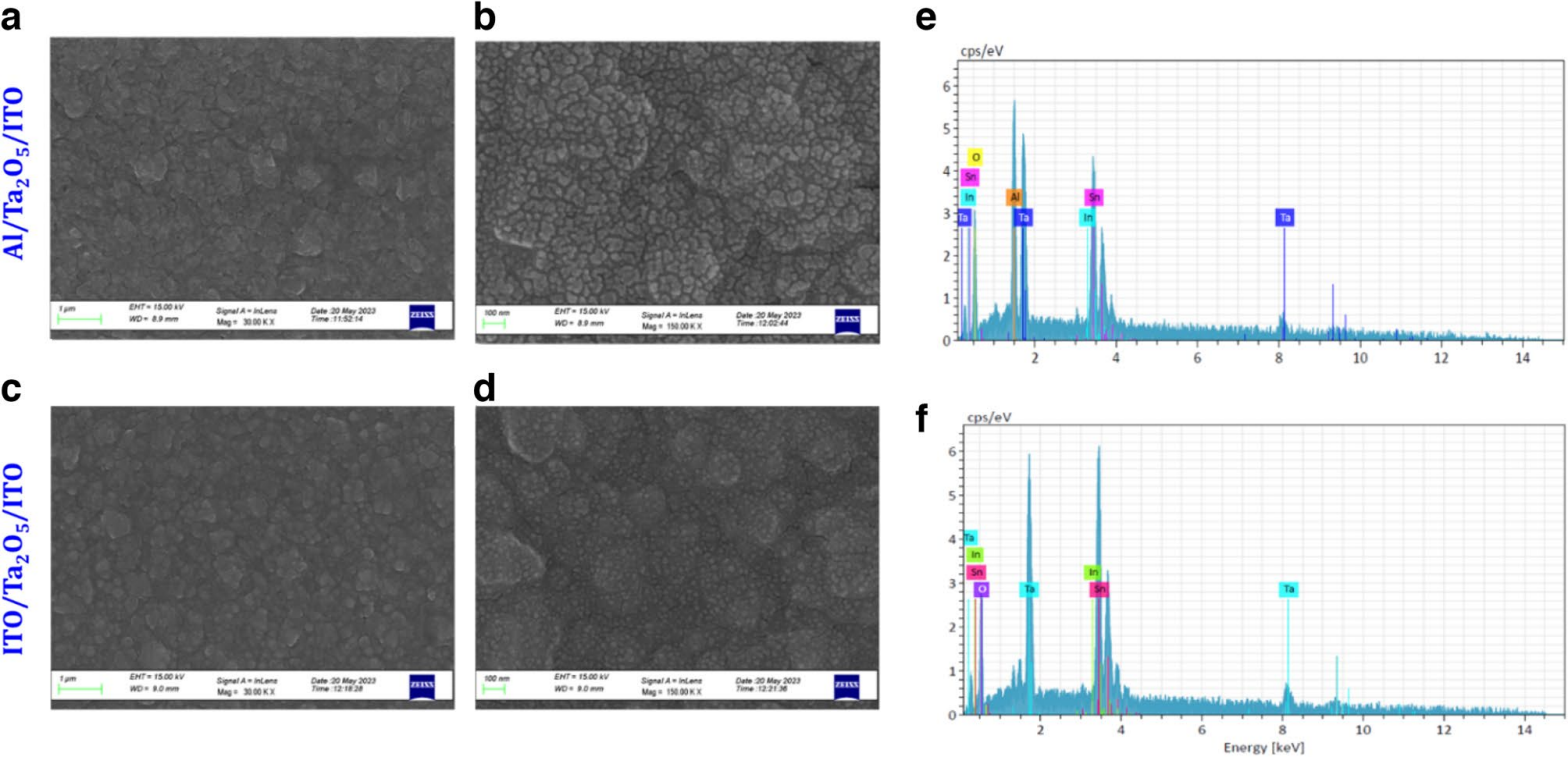
(**a**–**c**) FESEM topographic images of Al/Ta_2_O_5_/ITO and (**d**–**f**) ITO/Ta_2_O_5_/ITO at different magnifications.

The morphologies of these films were confirmed using field-emission-scanning-electron-microscope (FESEM) and the results are shown in Figs. 3a–d and 4a–f for two distinct thicknesses and top electrodes respectively. The top surfaces of these films clearly show a distinct morphology for two different thicknesses regime. The thinner films show smaller grain size of ~ 15–20 nm and thicker films show a larger grain size ~ 100–150 nm mentioned in Fig. 3b, which seems to be grown larger out of these smaller grains (visible from the fine structures), as the films gets thicker as also suggested by the root-mean-square (RMS) roughness value of these films ~ 1.5 nm is also found to be independent of the thickness. The similar topographic surface was also observed with top electrode layer that confirms layer-by-layer smooth growth. Also, no major defects such as macroparticles and pinholes are seen on the surface of devices. These devices were also observed under optical microscope and scanning electron microscope before to rule out any major deformity on the sample surface (as shown in Fig. S2).

**Figure 5:**
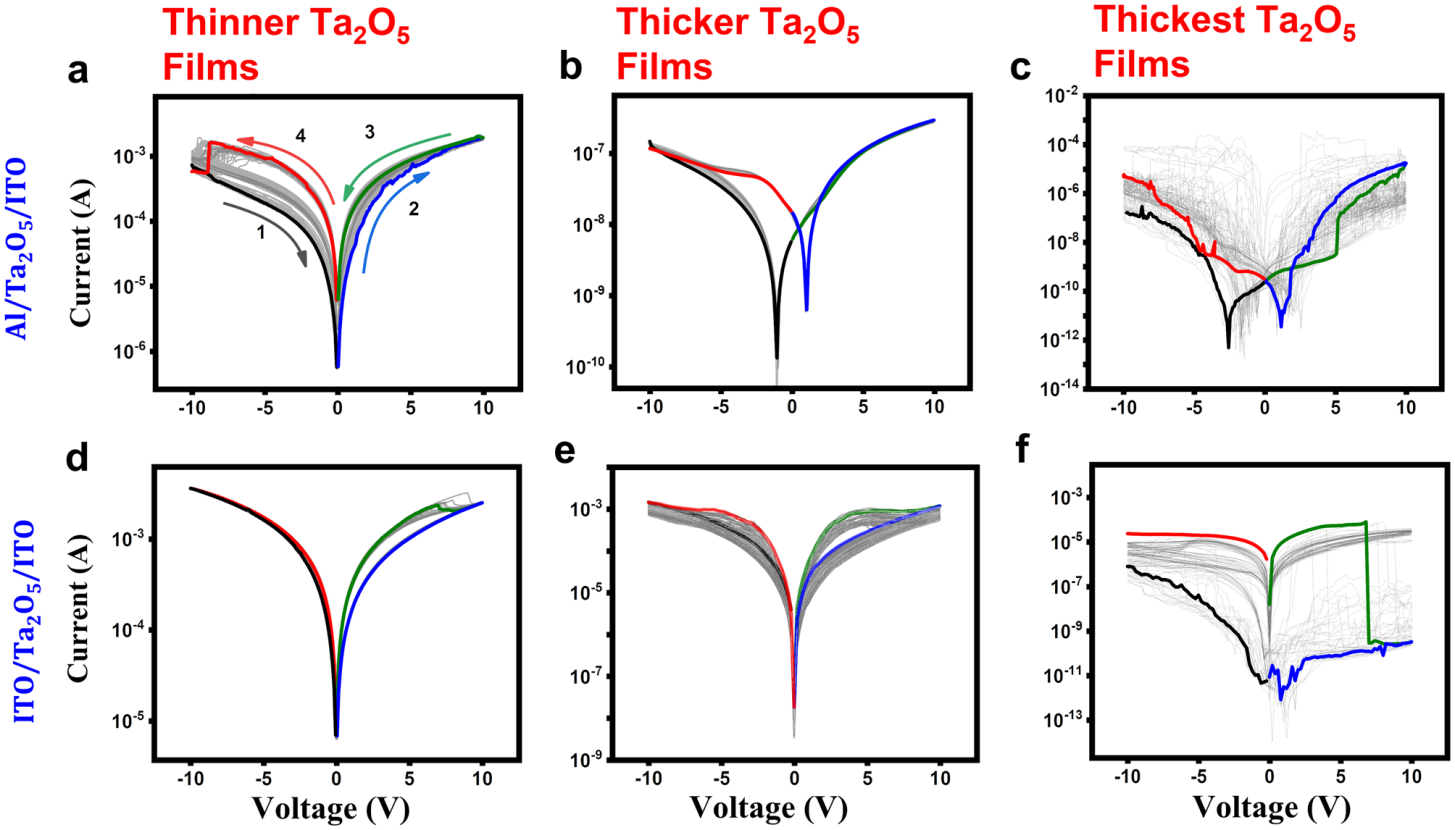
Resistive switching characteristics of asymmetric and symmetric devices with three distinct thicknesses of Ta_2_O_5_ film (~ 20 nm, ~ 80 nm, ~ 270 nm). Continuous 50 current–voltage (I–V) sweeps, highlighted 1st sweep.

The current–voltage (I–V) characteristics of asymmetric (Al/Ta_2_O_5_/ITO) and symmetric (ITO/Ta_2_O_5_/ITO) RS devices are measured in current-perpendicular-to-plane geometry by changing the bias voltage from 0 V → -10 V → 0 V →  + 10 V → 0 V → -10 V → 0 V in a triangular sweep. The bottom contact on ITO was grounded and the top contact is connected to on the top electrode while taking the measurements. Repeated datasets were taken at different locations to check the overall statistical behavior of the device. Figure 5a–f shows the analysis of 50 continuous cycles of I–V curves (plotted on log Y-axis) for three Ta_2_O_5_ film thickness of ~ 20, ~ 80 nm and ~ 270 nm of asymmetric and symmetric RS devices, respectively. The IV cycles show a clear hysteresis during the triangular sweep, indicating a nonvolatile bipolar resistive switching behavior. In these graphs, the high-resistive-state (HRS) and the low-resistive-states (LRS) are depicted by the black and red lines for the negative bias polarity, and the blue and green lines for positive bias polarity. After 50 cycles, there is no major change in the shape of I–V curves and the overall RS characteristic is retained, except the hysteresis get slightly narrower, which is expected in a typical memristive type behavior. Interesting, the overall shape of RS curves changes of asymmetric device as the film goes through a transition from thinner to thicker regime, as seen in Fig. 5a–f and Figs. S4 and S5. First, the minima in the currents are not centered around zero bias but observed around ± 0.2 V. Similar “butterfly shape” or ‘battery-like characteristics” of IV curves have been observed by many other groups in different materials such as Pt/MoO_*x*_/ITO^51^, other metal-oxides earlier due to internal built-in potential and non-Faradaic capacitive (NFC) capacitive effects^29^. Clearly, as the film thickness goes through a transition, the battery like characteristics are well defined and the overall operating current and voltages are significantly reduced, giving the operating power in the range of ~ 20–100 nW depending on the nature of top metal electrode, which is lower compared to other recent studies^14,30,35^. Thickest films (Fig. 5c,f) on the other hand become unstable and showed a random behavior for both electrodes.

**Figure 6:**
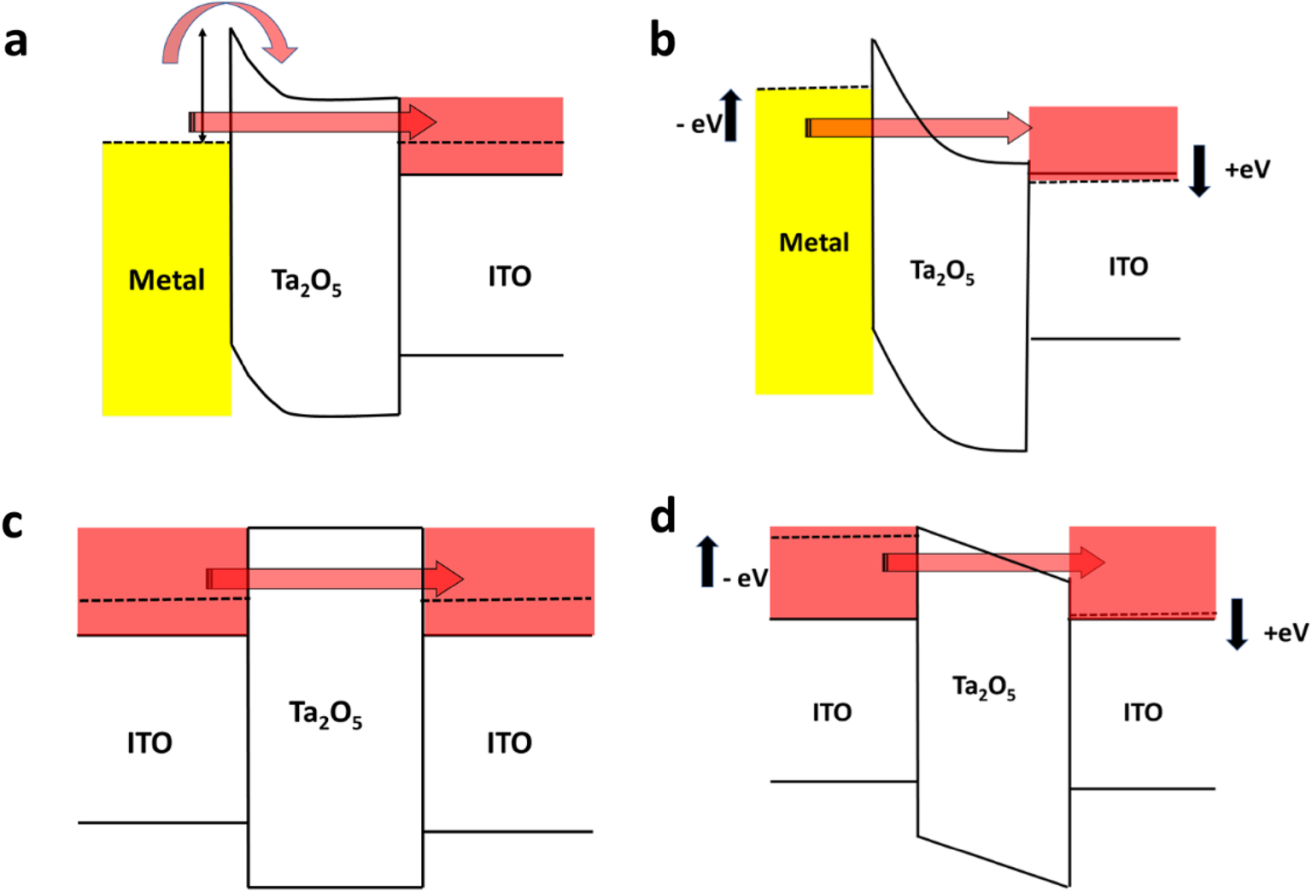
The schematic of energy band-diagram of (**a**,**b**) asymmetric device under zero-bias and biased conditions and (**c**,**d**) symmetric device under zero-bias and biased conditions.

Although the hysteresis is weak at one of the voltage polarities (positive for Al and negative for ITO), but an opposite polarity is always needed to completely reset the device. The preferential polarity could be due to the work function mismatch between the Al and ITO (Fig. S3). Also, the current range is also dependent on nature top electrode and the voltage polarity. These results can be explained using the band-diagram shown in Fig. 6a–d. The band diagram shows the respective positions of the Fermi levels, conduction band, and valence band of the top layers (Al, ITO), the intermediate layer Ta2O5, and the bottom layer ITO. In an asymmetric device (Fig. 6a), the Fermi level of the top electrode (Al) lies below the conduction band position of Ta2O5, but the Fermi level of ITO (degenerate semiconductor) is almost at the same level as the conduction band position of Ta2O5. So, under zero bias conditions (Fig. 6a), the electrons are expected to flow from Ta2O5 to Al but not from Ta2O5 to ITO, leading to a charge depletion at the Ta2O5/Al interface (band-bending). Therefore, a higher voltage is needed to overcome this barrier for tunneling (at higher bias) from Al to Ta2O5 as represented in Fig. 6b under biased condition. This observation is consistent with battery-like characteristics, as this barrier can contribute to an additional NFC. On the other hand, in a symmetric device (Fig. 6c,d), such a barrier is not formed at the interface, resulting higher current being observed at low bias voltages. In the case of the thickest film, this barrier does not play a significant role in overall direct or Fowler–Nordheim tunneling. Instead, the current is primarily dominated by bulk transport through Ta_2_O5 layer, which is heavily influenced by film defects. Consequently, no consistent Resistance Switching (RS) behavior has been observed. It is important to note that the IV characteristics for both thinner and thicker films are similar, except for large jumps and fluctuations in the hysteresis. Even the battery like characteristics could be observed. These large jumps and hysteresis could be attributed to bulk transport dominated by the conduction of through defects, in contrast to the tunneling mechanism observed for thinner and intermediate thicker films. The higher current observed in Fig. 5b,e can be attributed to the high degenerate nature of ITO and its respective Fermi level position with respect to the Ta_2_O_5_ band position. It should be noted that even in the asymmetric device, the higher current for the positive electrode is always observed, where the electrons transport from the bottom ITO to Ta2O5. This explains the higher asymmetricity in current (towards the positive bias) in asymmetric devices.

**Figure 7:**
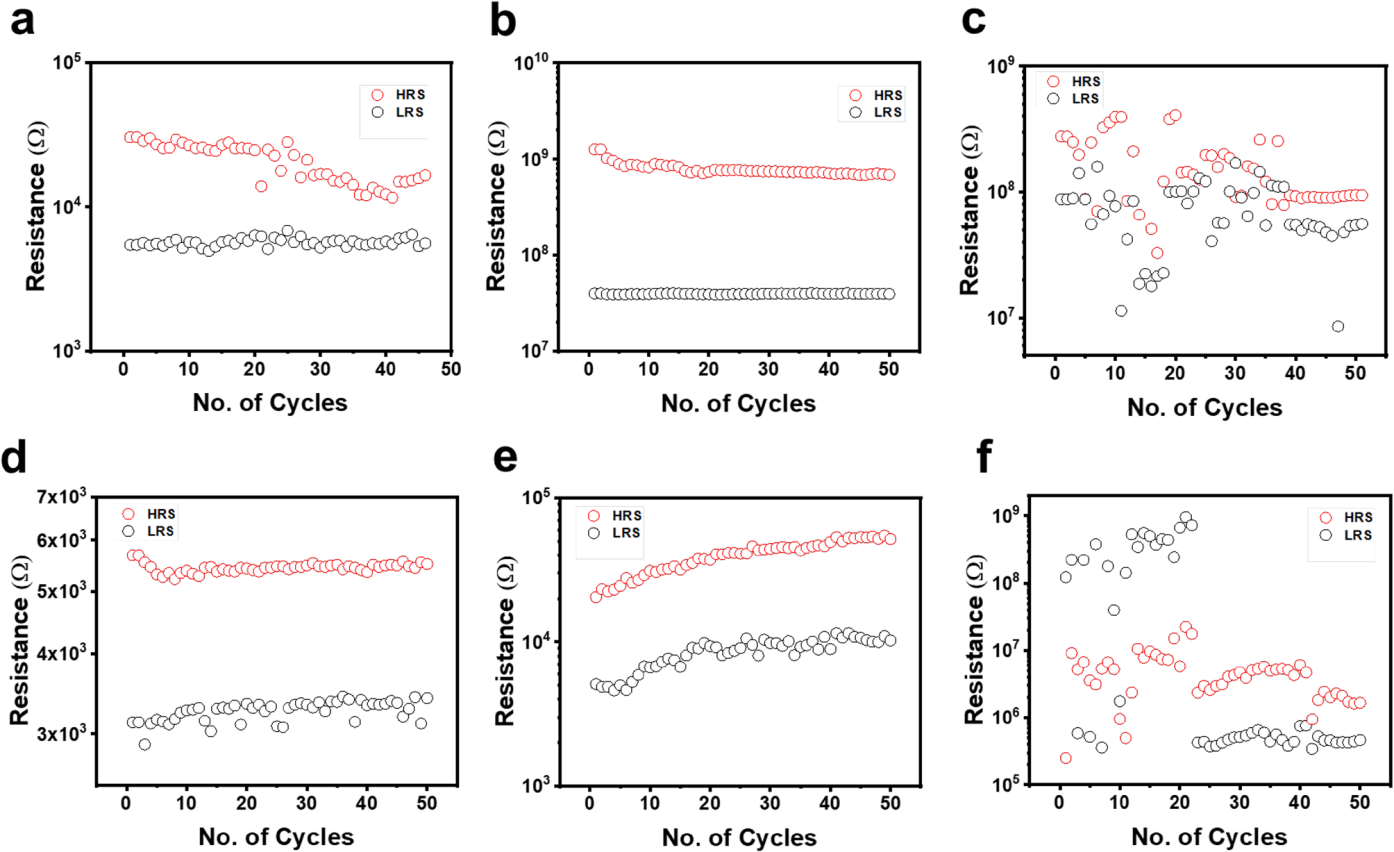
Endurance property of asymmetric and symmetric devices with three distinct thicknesses of Ta_2_O_5_ film (~ 20 nm, ~ 80 nm, ~ 270 nm) of continuous 50 cycles.

**Figure 8:**
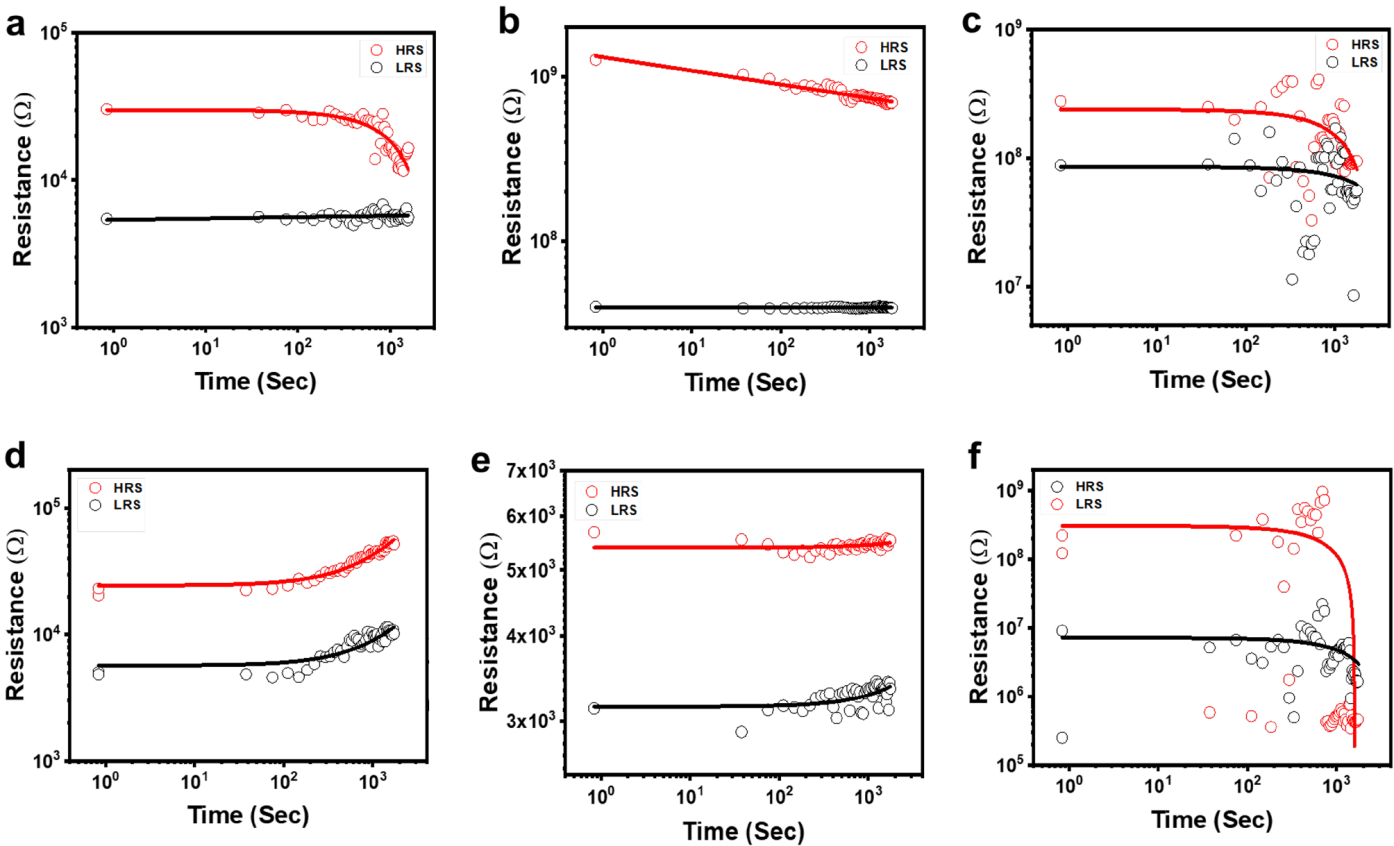
Retention property of asymmetric and symmetric devices with two distinct thicknesses of Ta_2_O_5_ film (~ 20 nm, ~ 80 nm, ~ 270 nm) of 10^3^ cycles.

Their endurance and retention of these devices are shown in Figs. 7 and 8 respectively. It has been observed that the films showing the battery-like characteristics relatively showed enhanced endurance and retention (Figs. 7b, 8b) compared to other films (Figs. 7a,c, 8a,c), as also reflected from the lower values of σ/μ extracted from the cumulative probability graphs shown in Fig. S6. Interestingly, thinner films showed better endurance and retention properties with ITO electrode.

**Figure 9:**
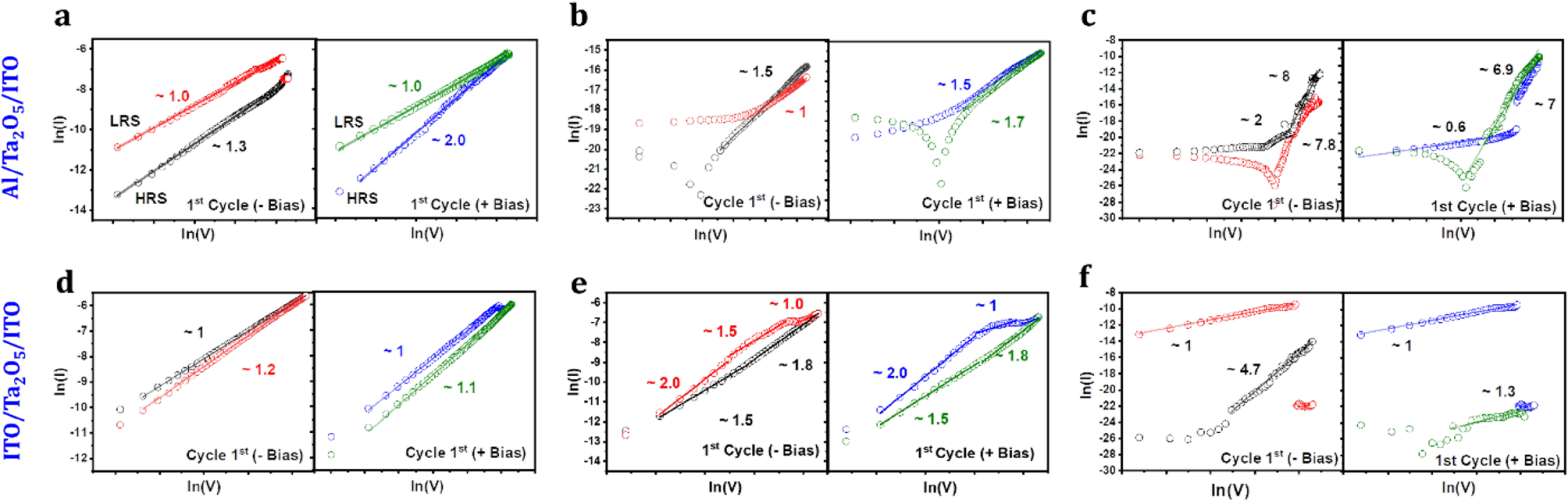
Linear fits between *ln (I) vs ln (V)* of resistive switching characteristics of asymmetric and symmetric devices for three distinct thicknesses of Ta_2_O_5_ (~ 20 nm, ~ 80 nm, ~ 270 nm).

To gain the understanding of the mechanism, these curves were linear fitted with different conduction mechanisms as per the procedure mentioned elsewhere^13^. The I–V curves in Fig. 5 could be the linear fitted with ln (I) vs. ln (V) as shown in Fig. 9, giving the slope values between ~ 1 and 2 that confirms the direct tunneling or defects assisted tunneling [space-charge-limited-conduction (SCLC)] is the dominant conduction mechanism. The direct tunneling from bottom to top electrode is not feasible for such a larger thickness. Therefore, the formation of filamentary path can also be concurrent mechanism with the trap-assisted tunneling. The weak asymmetricity in I–V curves is caused by the asymmetric nature of tunneling barrier due to the different work functions of electrode and insulator^13,52–55^. Compared to ITO as a top electrode, the Al and Ni coated metal tips (Supplementary Fig. S5) showed the battery-like characteristic, which is expected as these metals are more likely to participate in the redox reactions and change the local surface chemistry due higher oxygen affinity^56^ during the filament formation. On the other hand, thinner films do not have enough capacitance to hold the charge and the leakage current can kill the battery-like characteristics.

**Figure 10:**
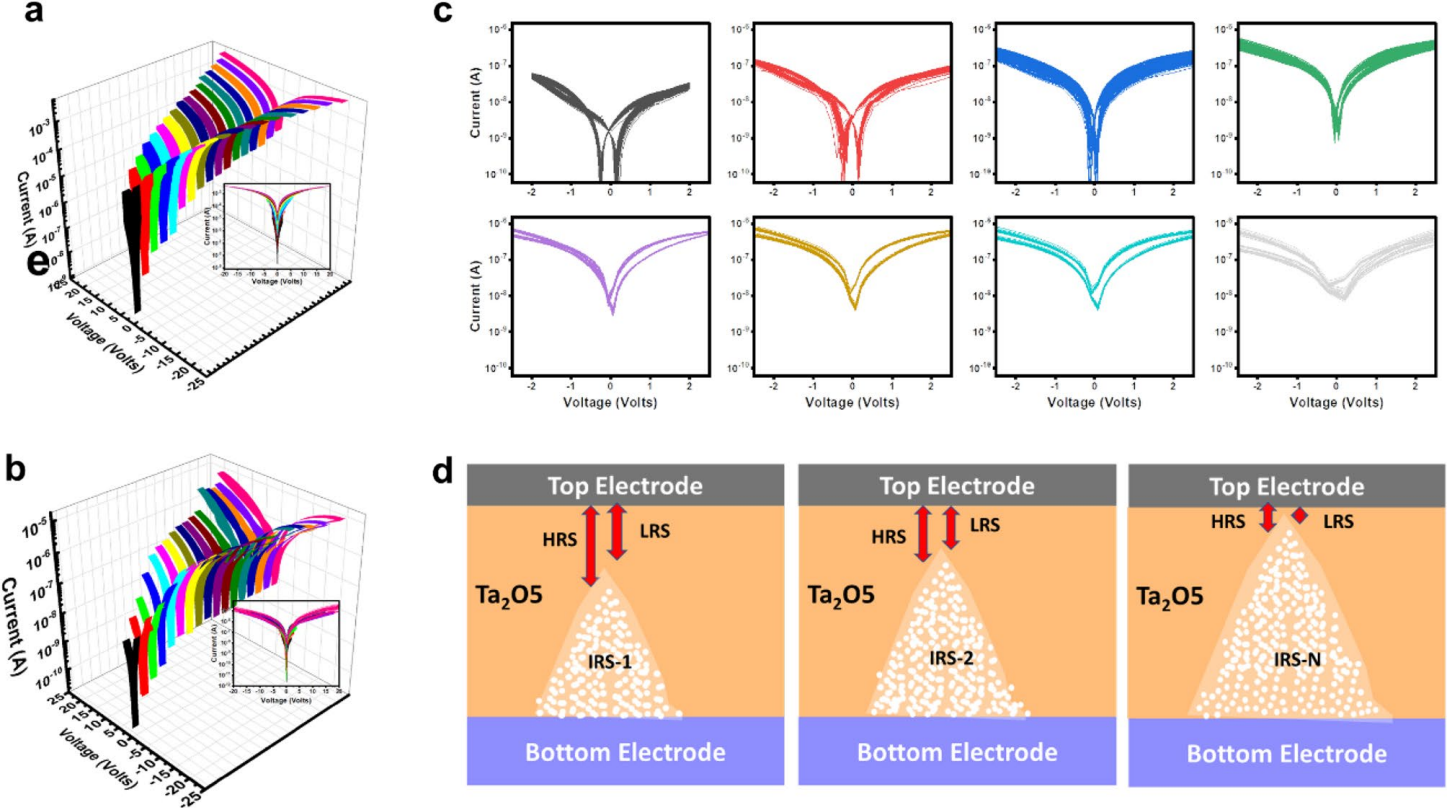
(**a**,**b**) I–V curves with increasing operating voltage and compliance current (**c**) the zoomed graphs showing the two current minima (in thicker films) getting closer, after increasing the range operation voltage and compliance current. (**d**) The schematic diagram explaining multiple intermediate-resistive-states (IRS) states and the low/high resistive states (LRS/HRS) tuned around each IRS through the modulation of filaments.

To further probe these effects, these films were also measured by increasing the range of applied voltage and the compliance current to see the role of Joule heating^17^. The overall I–V characteristic and ZBC is shifted upwards every time the range of sweeping voltage is increased, representing the multiple intermediate-resistive-states (IRS), as shown in Fig. 10a–c. Similar behavior could also be seen when the range of sweeping voltage is fixed, and the external temperature is increased (as shown in the Supplementary Fig. S7), which further confirms Joule heating through voltage/current pulses^17^. Interestingly, each IRS states can still be tuned between LRS and HRS states. We explain the mechanism through the help of a schematic diagram shown in Fig. 10d, based on the *in-situ* transmission electron microscopy imaging by other groups^57^. It is illustrated that different IRS states can be stabilized due to formation of different filaments having specific lengths and widths, depending on the range of operation voltage and compliance current. The voltage sweep causes the oxygen vacancies to migrate and forms the metallic filaments of Ta, TaO and TaO_2_ phases^34,57^. However, the LRS and HRS can still be tuned around each IRS through controlling the tunneling current by modulation in the filament lengths. It is important to note in Fig. 10c, each time the range of operation voltage and compliance current is increased, the battery-like characteristics gets weaker which could be due to fact that the formation of larger metallic filaments within the insulating matrix^57^ can effectively decrease the NFC, and hence weaken the total internal field.

In conclusion, we have used the tantalum oxide thickness, top electrode material and Joule heating through voltage and compliance current as controlling parameter to tune the overall RS characteristics in a highly transparent a-Ta_2_O_5_ and a-ITO thin films. Two distinct a-Ta_2_O_5_ thicknesses give completely different shapes of I–V characteristics. Thinner films showed a conventional RS behavior, while thicker films showed a battery-like characteristic. The RS of the device is explained by the filamentary type of mechanism where the low and high resistive states can be tuned through the modulations in filaments lengths and width. In thicker films, compared to ITO as electrode the pure metals showed the battery-like characteristic due to higher oxygen affinity for redox reactions and Faradaic capacitive effects. This study also opens new opportunities for understanding the battery-like characteristics in thicker films and incorporation of nanobattery concepts in tantalum oxide based memristors and RS devices.

### Supplementary Information

Supplementary Figures.

## Data Availability

All data generated or analysed during this study are included in this published article and its supplementary information files.
