# Supplementary material for: Resistive switching and battery-like characteristics in highly transparent Ta2O5/ITO thin-films
Source: Sci Rep. 2023 Aug 31;13:14297. doi: 10.1038/s41598-023-40891-2 (PMC10471767; doi:10.1038/s41598-023-40891-2)
Supplement: Supplementary file 1 — Supplementary Figures. [file 41598_2023_40891_MOESM1_ESM.docx]

**Supplementary**

**Resistive Switching and Battery-Like Characteristics in Highly Transparent Ta_2_O_5_­/ITO Thin-Films**

Darshika Khone^1^, Sandeep Kumar^2^, Mohammad Balal^3^, Sudipta Roy Barman^3^, Sunil Kumar^2^, Abhimanyu Singh Rana^1*^

*1 Centre for Advanced Materials and Devices, School of Engineering & Technology, BML Munjal University, Gurgaon 122413, India*

*2 Department of Physics, Indian Institute of Technology Delhi, New Delhi 110016, India*

*3 UGC-DAE Consortium for Scientific Research,* *Indore 452001, India*

**Corresponding author: rana.abhimanyu@gmail.com*


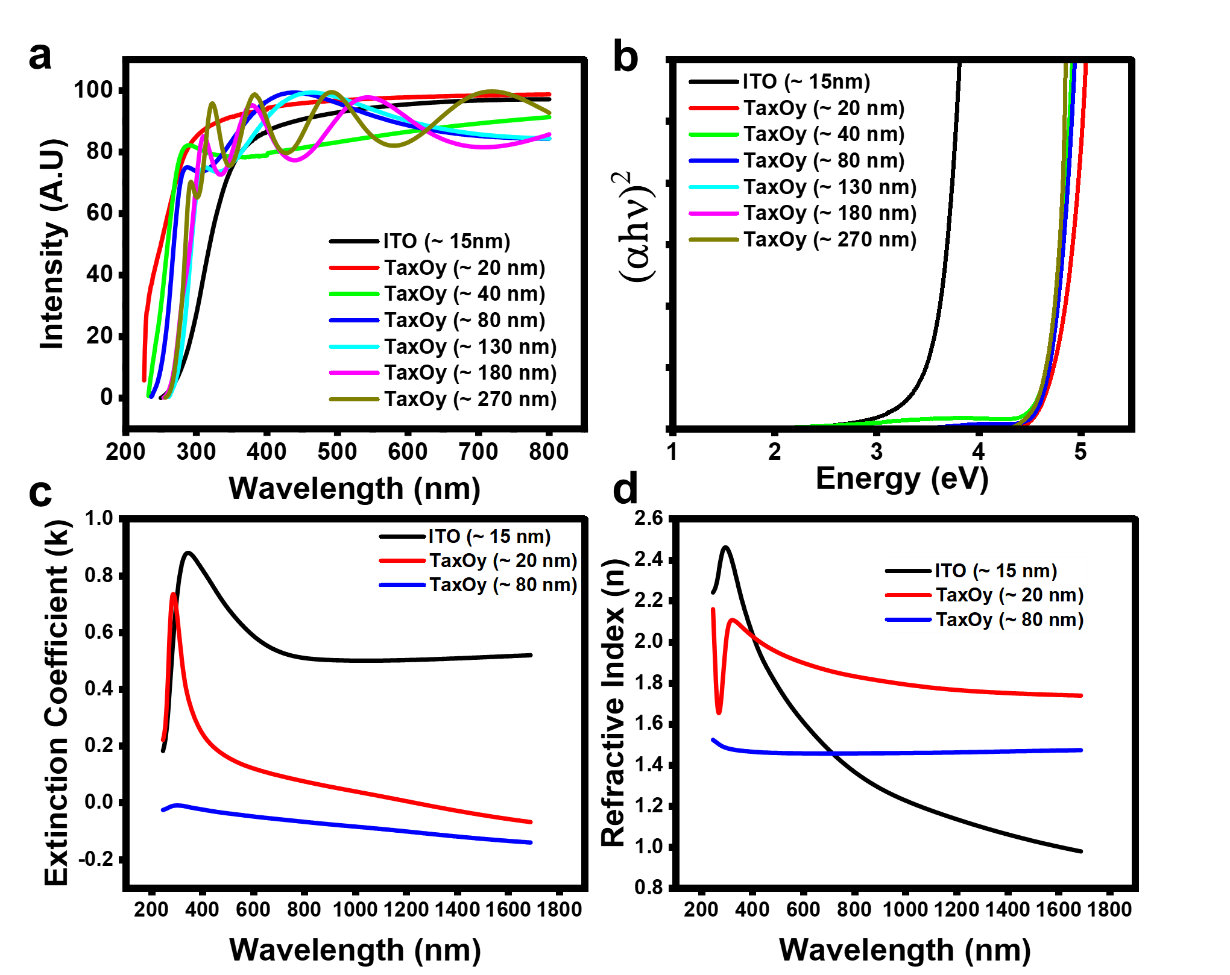


**Figure S1**: (a) UV-Vis. Transmission vs Wavelength of individual layers, (b) Tauc plots to extract the bandgaps of individual layers, (c-d) Plot of the refractive index (n) and refractive index (k) versus wavelength (λ).

**
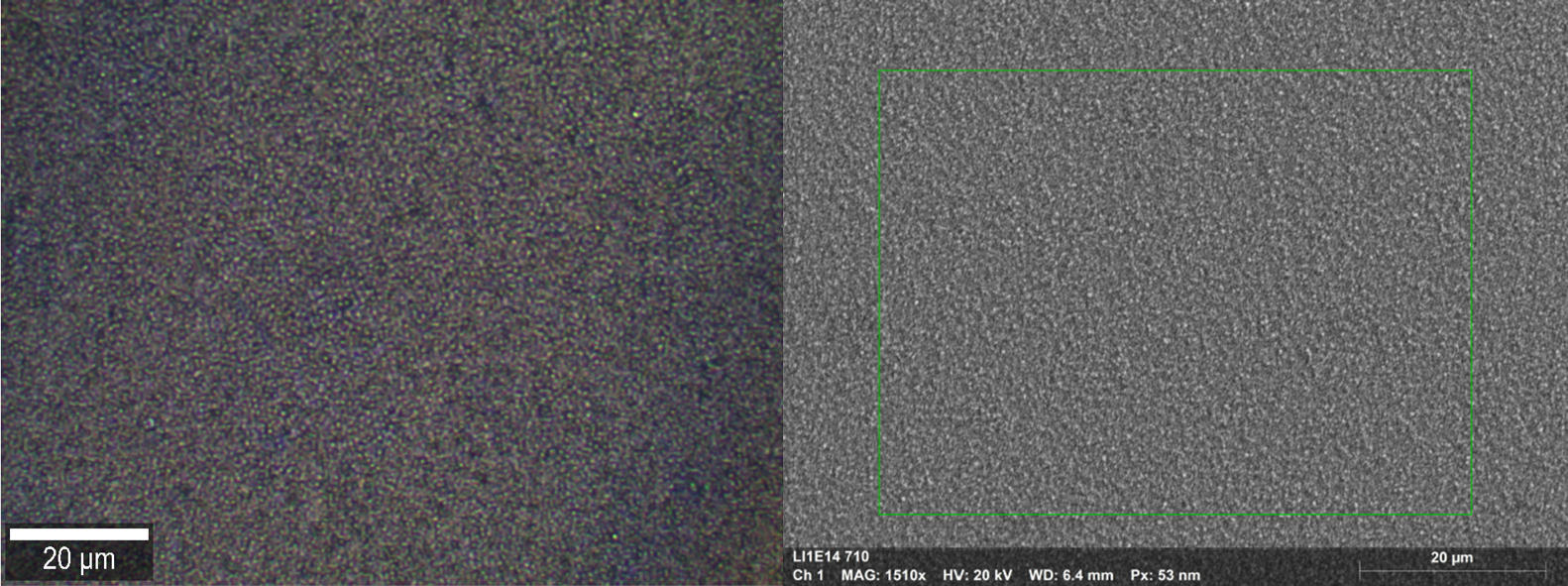
**

**Figure S2;** (a) Optical and SEM image of device at a large scale to rule out any major deformity and macroparticles.


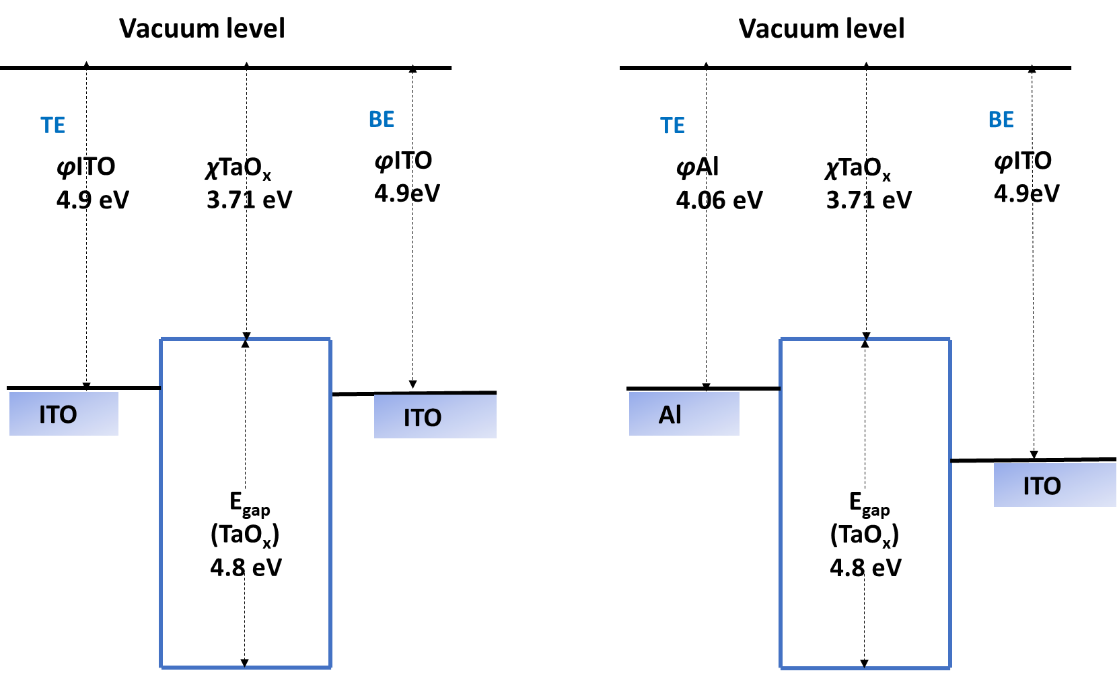


**Figure S3:** Relative positions of workfunctions of different electrodes from vacuum level and barrier heights of insulating layer.

1

**
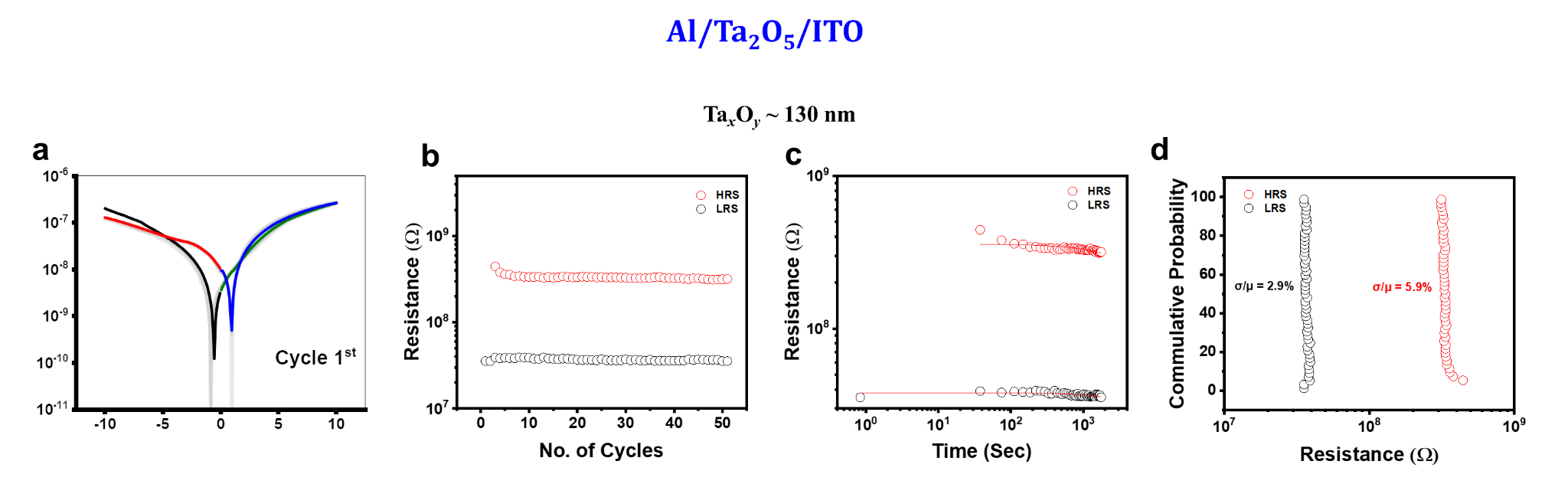
**

**Figure S4**: (a-b) Resistive switching characteristics of Ta_2_O_5_ film thickness ~ 130 nm.

**
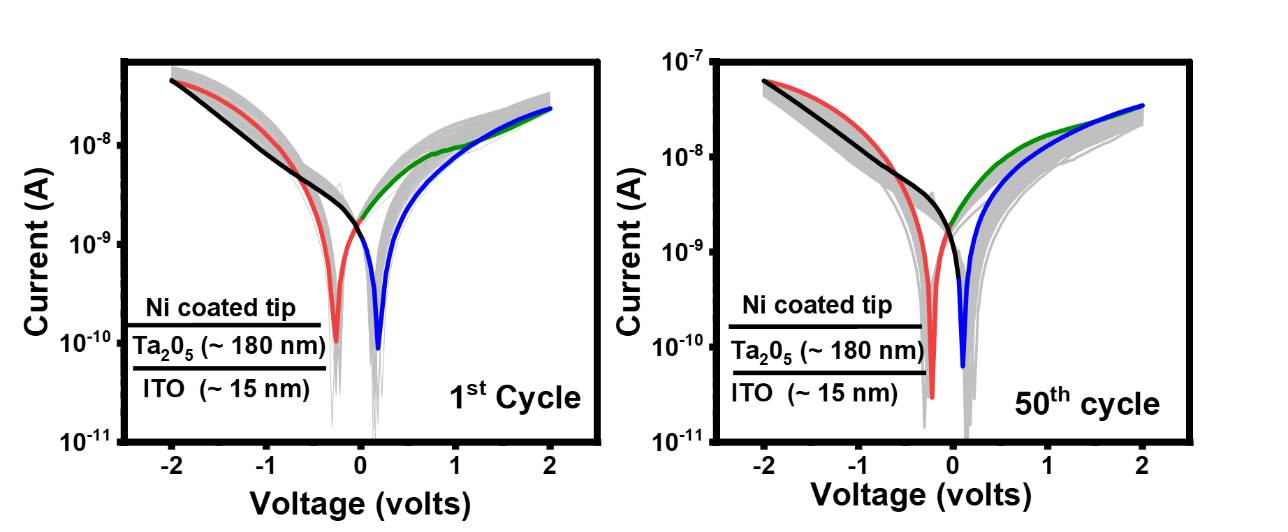
**

**Figure S5:** Resistive switching characteristics obtained using the Nickel coated metal tip (~0.3 mm radius)


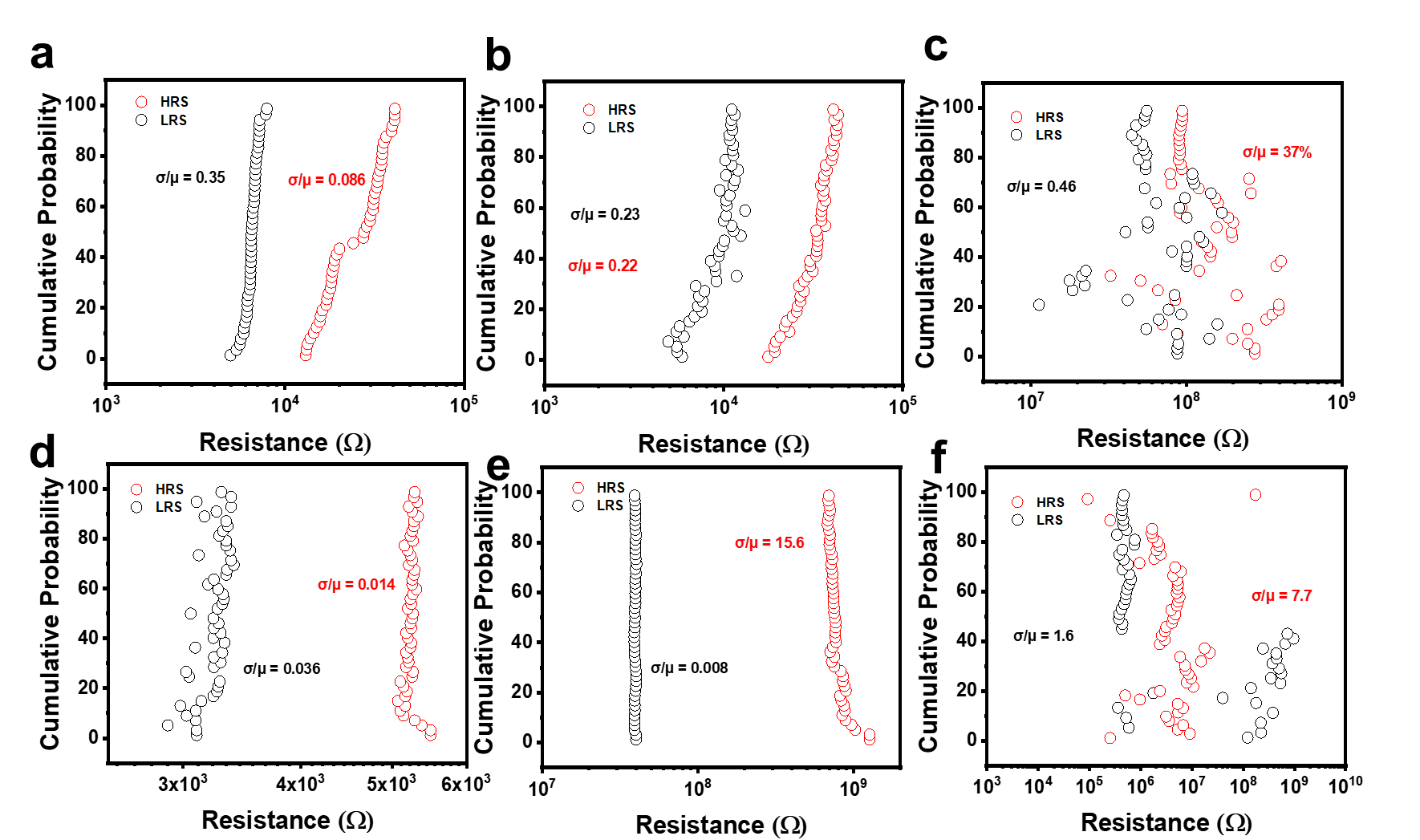


**Figure S6:** Cumulative probability of asymmetric and symmetric devices with three distinct thicknesses of Ta_2_O_5_ film (~ 20 nm, ~ 80 nm, ~ 270 nm).

**
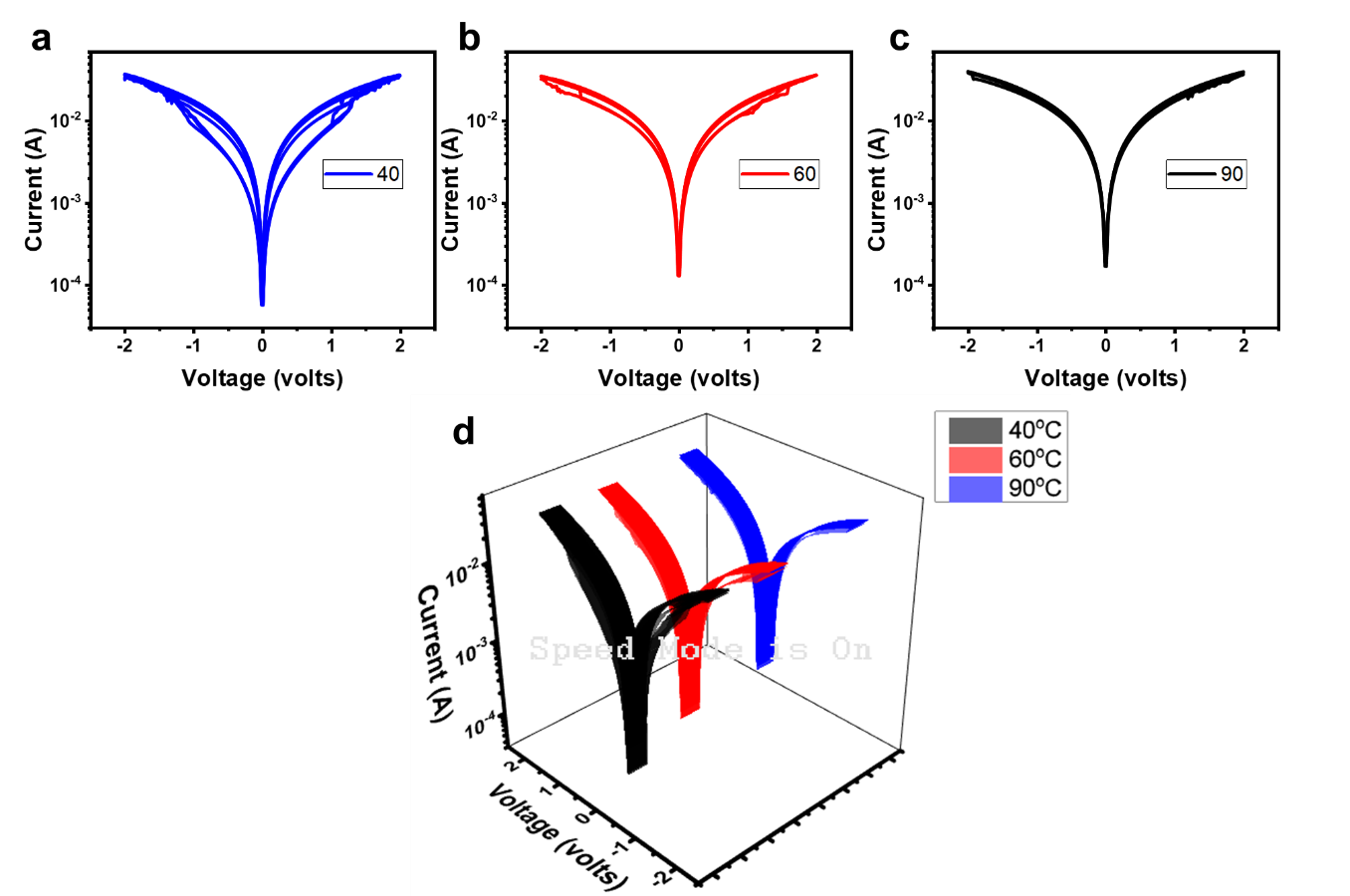
**

**Figure S7:** (a-c) Switching curves recorded at different temperatures ranges from 40 to 90 degree (d) 3D figure of RS curves.

1
